# “I think everybody should take it if they’re doing drugs, doing heroin, or having sex for money”: a qualitative study exploring perceptions of pre-exposure prophylaxis among female participants in an opioid intervention court program

**DOI:** 10.1186/s13011-020-00331-0

**Published:** 2020-11-23

**Authors:** Sarahmona M. Przybyla, Catherine Cerulli, Jacob Bleasdale, Kennethea Wilson, Melissa Hordes, Nabila El-Bassel, Diane S. Morse

**Affiliations:** 1 Department of Community Health and Health, School of Public Health and Health Professions, University at Buffalo, 3435 Main Street, 305 Kimball Tower, Buffalo, NY 14214 USA; 2 Department of Psychiatry and Medicine, University of Rochester, 601 Elmwood Avenue, Rochester, NY 14642 USA; 3 Susan B. Anthony Center, University of Rochester, PO Box 270435, Rochester, NY 14627 USA; 4 School of Social Work, Columbia University, 1255 Amsterdam Ave, New York, NY 10027 USA

**Keywords:** Pre-exposure prophylaxis (PrEP), HIV prevention, Opioid use disorder, Qualitative research, Opioid court, Drug court

## Abstract

**Background:**

Women’s rise in opioid use disorder has increased their presence in the criminal justice system and related risk behaviors for HIV infection. Although pre-exposure prophylaxis (PrEP) is an effective biomedical HIV prevention treatment, uptake among this high-risk population has been particularly low. Considerably little is known about the interplay between justice-involved women with opioid use disorder and HIV prevention. The aim of this study was to explore PrEP knowledge, attitudes, and perceptions for personal and partner use among women participants in the nation’s first ever opioid intervention court program.

**Methods:**

The authors conducted semi-structured, in-depth interviews with 31 women recruited from an Opioid Intervention Court, a recent fast-track treatment response to combat overdose deaths. We utilized a consensual qualitative research approach to explore attitudes, perceptions, and preferences about PrEP from women at risk for HIV transmission via sexual and drug-related behavior and used thematic analysis methods to code and interpret the data.

**Results:**

PrEP interest and motivation were impacted by various factors influencing the decision to consider PrEP initiation or comfort with partner use. Three primary themes emerged: HIV risk perceptions, barriers and facilitators to personal PrEP utilization, and perspectives on PrEP use by sexual partners.

**Conclusions:**

Findings suggest courts may provide a venue to offer women PrEP education and HIV risk assessments. Study findings inform public health, substance use, and criminal justice research and practice with justice-involved participants experiencing opioid use disorder on the development of gender-specific PrEP interventions with the ultimate goal of reducing HIV incidence.

## Background

In the United States, life expectancy has declined since 2014, principally propelled by the opioid epidemic [1]. In 2017, synthetic opioids were the primary driver of the nearly 70,000 drug-related overdose deaths [2, 3]. Women are not immune to this public health emergency; the crude rate of drug overdose deaths among women between the ages of 30–65 rose by 260% from 1999 to 2017. Of significance, overdose death rates among women grew by 1643 and 915% for synthetic opioids and heroin, respectively, in the same timeframe [4]. Currently, more than 2 million Americans experience opioid use disorder (OUD), including more than 1.7 million individuals with a prescription pain reliever use disorder and 500,000 individuals with a heroin use disorder [5]. In addition, opioid misuse (defined as the misuse of prescription pain relievers or the use of heroin) is common, with approximately 10.3 million individuals reporting past-year opioid misuse [5].

Public health, medical researchers and practitioners are recognizing the significant infectious disease consequences of the opioid epidemic. Injection drug use (IDU) is contributing to the increase in viral hepatitis infections and nearly 10% of all incident HIV infections are among people who inject drugs (PWID) [6]. Although the United States has reduced the rate of HIV infections attributable to IDU by nearly half (48%) between 2008 and 2014 [7], 2015 marked the first year in which the number of IDU-attributable HIV infections rose. This increase was directly connected to an HIV outbreak in one rural community in Indiana, in which new HIV infections were traceable to injection use of oxymorphone [8]. In October 2020, the Centers for Disease Control and Prevention released a health advisory regarding HIV outbreaks among PWID, notifying clinical and public health service providers about the growing prospect of new HIV clusters [9].

Nearly one in every five HIV diagnoses nationally occur among women. Despite declines among most racial/ethnic groups, trends from 2010 through 2017 show that HIV incidence has been relatively stable among non-Hispanic White women [10]. Similarly, while HIV diagnoses among PWID have declined over time, HIV incidence increased by 11% between 2016 and 2018 in this subpopulation, particularly among non-Hispanic Whites and those less than age 40 [11]**.** Given these patterns, there is an increasing public health concern that the opioid and HIV epidemic paths could converge to disrupt recent trends in decreased HIV incidence [12]**.**

Pre-exposure prophylaxis (PrEP) is an incredibly effective tool to prevent HIV infection [13, 14]. However, to maximize its prevention potential, PrEP uptake among sub-populations at risk of HIV acquisition is essential. Although PrEP utilization has grown substantially since it was approved by the US Food and Drug Administration (FDA) in 2012, PrEP awareness and access, among women and PWID in particular, are low [15–19]. Estimates indicate that nearly 170,000 women and 73,000 PWID are clinically eligible for PrEP under current FDA guidelines [20]; however, the medication is underutilized, specifically among women [21], as PrEP coverage is three times as high among men relative to women [22]. In addition, few evidence-based interventions exist to specifically promote PrEP uptake among women, particularly among those who use drugs [23, 24].

At first glance, the justice system is an unfamiliar and unexpected partner in the HIV prevention circle. Of note, the node of women’s rising representation in OUD is their increasing presence in the justice system and related risk behaviors for HIV infection. Given increased risk behaviors, such as transactional sex and injection drug use [25–27], justice-involved women shoulder a disproportionate burden of HIV infection [28, 29]. In addition to HIV, women with criminal-justice involvement also experience comorbid hepatitis C at rates higher than their male counterparts [30]. Consequently**,** the justice system may be an innovative point of intervention to jointly tackle OUD and HIV using a dual-pronged approach. To date, few studies have qualitatively examined HIV prevention, including PrEP, from the perspective of female opioid users within the justice system. As such, our research aim was to gain a greater understanding of PrEP knowledge, attitudes, and perceptions for personal and partner use among female participants with OUD in an opioid intervention court program.

## Methods

### Participants and procedures

The study venue was the Buffalo Opioid Intervention Court (Court). The first of its kind in the nation beginning in May 2017, this specialized drug court provides immediate, intensive intervention for participants at high risk of opioid overdose, including medication-assisted treatment within 24 h of arrest and three consecutive months of court monitoring. A research assistant recruited study participants in the courthouse lobby outside the courtroom from January–July 2019. Because the research assistant attended Court and had a copy of the docket, she knew who was in the Court track of the Drug Treatment Court and only approached those particular women. According to court records, there are approximately 110 people in the Court at any given time; 40% were women (Opioid Intervention Court staff, personal communication, July 17, 2018). The research assistant approached women, verbally confirmed they were Court participants, and informed them that she was part of a team conducting a research study. Due to the heightened risk of coercion for individuals within a justice system involvement program, we advised women that their participation and content of their research involvement were confidential (including to Court staff) and had no bearing on their legal status.

Research staff provided information about study procedures and screened interested women for eligibility (i.e., age 18 years or older, able to speak in English, identified as female, and Court program participants). Eligible, consenting women self-administered two questionnaires and participated in a subsequent qualitative interview. The team recruited 42 eligible women all of whom agreed to participate. Eleven women failed to complete an interview, most commonly due to incarceration (*n* = 3), entrance into a long-term rehabilitation program (*n* = 2), or relocation to another city or state (n = 2). Between January–August 2019, we conducted 31 interviews until we reached content saturation with no new emergent themes. The questionnaires included items regarding demographic characteristics (e.g., age, employment) and health history (e.g., testing and diagnosis of bacterial sexually transmitted infections, HIV, and hepatitis C). We did not confirm these self-reports with any medical or criminal justice records. The private semi-structured interview covered a range of topics such as general awareness of the concept of PrEP for HIV prevention, how participants envisioned themselves using PrEP, and their perspectives on sexual partner use of PrEP. The interviews lasted an average of 45 min and took place at either a university research office or a local community justice center within walking distance of the courthouse. The study reimbursed participants a total of $60 in cash ($20 for the questionnaires and $40 for the interview) and four round-trip public transit passes. With participants’ consent, we digitally audio-recorded all interviews and then a professional transcriptionist created Microsoft™ Word documents which we de-identified.

The first three interviews took place with real-time team observation via Zoom™. The team met on subsequent days to provide feedback until interviews were conducted according to team standards. The interviewer conducted the remaining sessions alone, with senior team members periodically listening to random portions of the interviews for fidelity and to assess saturation. As per our design, after the first three interviews, we refined the interview processes and adapted interview questions to ensure that our research aims were being met, using consistent interview protocols [31]. The study protocol received Institutional Review Board approval from the two participating research universities affiliated with the study.

### Analysis plan

The multidisciplinary team (including trained graduate research assistants) analyzed the data by conducting a Consensual Qualitative Research analysis, an integrative approach which incorporates elements from phenomenological, grounded theory, and comprehensive process analyses [31]. Research team members understood meaning through the words of the text, staying close to the data and avoiding over-interpretation. We used a consensus process to arrive at a common understanding regarding the meaning of the data.

The team jointly reviewed the first three transcriptions to create a preliminary list of primary and secondary domains or codes to group the data. As this was an iterative process, the team met repeatedly to share insights, reflect on disagreements, and reach consensus. We continued to meet in smaller teams after the next 11 interviews were completed and transcribed to review the codebook and revise the preliminary list of codes. We co-created the final codebook; we then recoded the first set of transcripts with the final codebook (at least two people per transcript). For this paper, the team performed a final cross-analysis to construct common themes. We focused specifically on codes and overarching themes relating to HIV transmission and prevention. If a quote within a transcript was considered illustrative of a specific point, we noted the quotation page and lines. We could assign specific quotes one or more codes based on content related to participants’ experiences and relevant to our overarching research questions. The team asked two formerly incarcerated women to review our results and conclusions to have respondent verification of its trustworthiness [32].

## Results

### Participant characteristics

We provide demographic characteristics of the study sample in Table 1. The average age was 30.9 years (SD = 6.8), 9.7% identified as Latina, and all women were cisgender. Most women were unemployed (71%) and reported stable housing (94%). In terms of sexual activity, 55% reported sex with men and 25.8% reported sex with men and women. While 71% of the sample reported being aware of PrEP, knowledge regarding its use, efficacy, and availability was limited. No participants reported past or current PrEP use. In terms of HIV, hepatitis C, and sexually transmitted infection care, virtually all women reported lifetime testing experiences. More than two-thirds of women reported a diagnosis of hepatitis C and more than one-third reported at least one bacterial sexually transmitted infection diagnosis in her lifetime (Table 2).

**Table 1:** Participant Characteristics (*N* = 31)

Characteristics	n (%)
**Age** [M (SD)]^a^	30.9 (6.8)
**Ethnicity**^b^	
Hispanic/Latina	3 (9.7)
Not Hispanic/Latina	28 (90.3)
**Educational Attainment**	
≤ GED or High School Diploma	21 (67.7)
**>** High School Diploma	10 (22.3)
**Employment Status**	
Full or Part Time	9 (29.0)
Unemployed	22 (71.0)
**Housing Status**	
Stable	29 (93.5)
Unstable	2 (6.5)
**Sexual Activity**	
Sex with Men	17 (54.9)
Sex with Women	1 (3.2)
Sex with Men and Women	8 (25.8)
Sexually Inactive	5 (16.1)

*Note*. ^a^*M* Mean, *SD* Standard deviation. ^b^Race excluded due to confidentiality concerns

**Table 2:** HIV, Hepatitis C, and Sexually Transmitted Infection Experiences (*N* = 31)

Characteristics	n (%)
**Hepatitis C**	
Testing	30 (96.8)
Diagnosis	21 (67.7)
Receipt of Treatment	16 (90.3)
**HIV**	
Testing	31 (100.0)
Diagnosis	1 (3.2)
Receipt of Treatment	0 (0.0)
**Bacterial Sexually Transmitted Infections**	22 (71.0)
Testing	31 (100.0)
Diagnosis	12 (38.7)
Receipt of Treatment	11 (35.5)

### Thematic findings

Three themes emerged from the qualitative interviews: HIV risk perceptions, barriers and facilitators to PrEP initiation, and perceptions of PrEP use by sexual partners (Table 3).

**Table 3:** Results from the Thematic Analysis of Participant Interview Data (N = 31)

Theme	Domain	Code
**HIV Risk Perceptions**	General sexual transmission risk perceptions	Mutual monogamy
		Sexual assault
		Discontinuation of sex work
		Sexual inactivity
	Transmission risk perceptions related to substance use	Discontinuation of substance use
**Barriers and Facilitators to PrEP Initiation**	Scenarios supporting PrEP uptake	Condomless sex with new or multiple partners
		Relapse or return to injection drug use
		Re-engagement in sex work
	Obstacles to PrEP uptake	Side effects
		Daily pill adherence
**Perceptions of PrEP Use by Sexual Partners**	Risk cues of partner PrEP use	Partner infidelity
		Partner engagement in high-risk sexual behaviors
		Partner engagement in high-risk substance use behaviors
	Concurrent PrEP use	Simultaneous PrEP use by participant and partner
		Mutual safety

### Theme 1: HIV risk perceptions

Participants reported various opinions on personal HIV risk perception in present-day terms and also in the hypothetical future. Overall, women reported low perceived risk for HIV transmission related to perceived mutual monogamy, discontinuation of sex work, general sexual inactivity, elimination of situations that would heighten the risk of sexual assault, or discontinuation of substance use. This perception had an inherent temporal ordering where women would describe their previous behaviors that increased their risk of HIV acquisition including sex work, sexual assault, multiple partners, condomless sex, sex while using drugs, and needle-sharing behaviors.

The majority of participants indicated that they are currently in a mutually monogamous relationship or not sexually active. Consequently, HIV risk perception was relatively low for most women at the time of the interview. As one participant shared, “Right now I’m not sexually active. I haven’t been sexually active in over a year. So, I don’t really pay [HIV] no mind.” (Participant 22, 45 years old). This represents a sentiment echoed by other women regarding their goal of sobriety coupled with being in a committed partnership: “[I’m not at risk for HIV] because I’m not using. And like I said, if I did use, I always used a new needle and I’ve been with the same person for a long time now.” (Participant 33, 28 years old).

While many participants self-assessed their HIV risk as low, others described scenarios that could change risk calculations. These conversations often focused on mistrust and dishonesty between sexual and needle-sharing partners that may elevate or reduce HIV risk perception based on their interpersonal encounters and social environments. As one woman describes:

Just that, I don’t know, risk versus reward. There’s no reward getting HIV. The risk is really high, so it’s just not worth it, and people lie all the time. You can’t count on someone to go and get tested and bring in the test results. (Participant 29, 30 years old).

Women described the idea of HIV-risk considerations being rooted in an informal, spur-of-the moment feeling that estimates a partner as potentially infected with HIV or not. In this case, women may erroneously judge a sexual or needle-sharing partner as low risk or rationalize a behavior in her mind. As one woman explains:

It’s not the safest lifestyle, but I know people who will share a needle with anybody and may not think twice about it. Okay, people lie all the time. Why would you trust somebody with something like that? But I would consider somebody like that a higher risk, but I know on paper, I would be considered in the high-risk category. (Participant 32, 29 years old).

Risk calculations in hindsight feel very different from those when women decide to engage in behaviors that may elevate their HIV risk. For example, one participant describes the idea of a partner’s HIV status not being blatantly obvious:

Even though I’m not using needles, it doesn’t mean that I won’t [share needles] because I do have slipups with them and even with sexual partners. You can’t look at somebody and tell that they have it and it could be somebody that you’d least expect. (Participant 25, 35 years old).

### Theme 2: barriers and facilitators to PrEP initiation

While the majority of participants were aware of PrEP prior to study involvement (71% according to baseline surveys), direct knowledge was low. Specifically, most women could not accurately describe the purpose of PrEP or express how the medication works. Some indicated a vague recollection of the concept; however, knowledge of its use for HIV-uninfected individuals was rare. Some participants believed it was synonymous with post-exposure prophylaxis. Others expressed disbelief and surprise that such a medication existed. As one participant stated:

I have no idea about a medicine that you can take to prevent HIV. I’ve heard of it, like here and there. People will tell me ‘I’m taking a medicine to prevent HIV’. I say, you’re crazy. Is that really a thing? (Participant 41, 29 years old).

For those women who had expressed prior awareness of PrEP, most described learning about it from either television commercials or print media at a local HIV service agency that offers a syringe exchange program and HIV testing services.

Participants shared perceived barriers and facilitators to personal PrEP uptake related to pill-taking behaviors and various risk scenarios that would motivate or impede their interest in PrEP use. Specifically, women described multiple scenarios that would facilitate PrEP initiation including condomless sex with new or multiple partners, relapse or return to injection drug use, and reengagement in sex work. In terms of sexual risk, women often reflected on the past and used hindsight to consider whether PrEP would have been appropriate for them. As explained by one participant:

I probably should have taken [PrEP] when I was using, when I was actively prostituting. I would have been huge to take it because ultimately the idea is to not expose yourself at all, but I was putting myself in high-risk situations and not having any backup. Ideally, I should have just been on it altogether. But when I started using again, it wasn’t necessarily my intention to go back to doing that. (Participant 38, 25 years old).

For other women looking prospectively, they acknowledge the role that PrEP may play in their future sexual risk behaviors, as described by one woman who shared:

I still do associate with people within a certain circle and you never know. I’m not going to say that I’m still not going to turn a trick here and there in order to get money. Why not protect myself? (Participant 35, 30 years old).

They expressed insight regarding the frequent course of substance use disorder including relapse and remission. Some women described PrEP as a representation of self-respect and self-care, especially in light of the potential for concomitant exposure to HIV via sexual and IDU behaviors. As one participant shared:

I hope I don’t, but let’s say just a few years down the road if I ever do relapse or something does happen. Or even if I don’t relapse and meet someone and something happens, I kind of want to be a little extra prepared … (Participant 19, 22 years old).

Other women could foresee a situation warranting PrEP use given a change in sexual partnerships. Many women did not envision themselves to need PrEP at this point in their lives, often related to their overall recovery process. They did not reflect upon the fact that one sexual encounter or shared needle could result in HIV acquisition. However, they often expressed a “what if” scenario where they could visualize a future in which PrEP played a role in their HIV prevention efforts, most often related to transactional sex. For example, one woman described her interest in PrEP with a hypothetical future:

I’m not perfect. Sometimes I do forget to wear protection. I’m human so I fall short, but I’m doing everything I can to prevent that from happening. Like PrEP–I don’t feel like I need it at this point. If I were to start up prostituting really bad or whatever it would definitely be something that I would want. (Participant 5, 28 years old).

This sentiment is echoed by another woman who relates her current versus future interest in PrEP utilization by stating:

Realistically, right now, if I don’t ever go back to using stuff, I wouldn’t need it, right? If I went back to using I know I would definitely need it, because I know I’m going to start prostituting again. It just–my pockets aren’t that deep. It just goes hand in hand. (Participant 7, 30 years old).

Commonly cited barriers to PrEP initiation included side effects, concerns about daily pill adherence, and overall impediments to use of the medication. For example, one woman describes her hesitation to initiate PrEP as, “I’d be concerned too about the side effects of it and personally I try not to take anything that I don’t need to. I know a lot of those things have side effects, so I’d wonder about that.” (Participant 21, 40 years old).

When prompted about what would make them want to take or not take PrEP, women often responded with apprehension about the need to take another daily medication. The regimen was considered too burdensome, as explained by one woman who states:

I’m on a medicine that I take every day. So, I’m not really a person that would want to take something every day. Either I’d forget it, or it’s just not … Like I’m not on psych meds for the same reason. I don’t believe in being on something for the rest of your life. I want to be off methadone as soon as I can, because I don’t want to take something every day. (Participant 14, 29 years old).

Another participant speaks paradoxically about potential PrEP use in the context of her recovery process by sharing:

I think if I were to take [PrEP], I would be using … But when I’m using, I’m not going to care about [taking PrEP]. So that would probably be the only time I would take that because it’s a daily thing. That’s like taking birth control. It’s just a lot to have to do something every day. Like with Suboxone it’s different because I know I need it. It’s the only thing I’m getting right now to satisfy my brain. That is in the back of my head thinking, well, is that the only thing? Even if I have it next to the Suboxone do I really want to take that? But then, I don’t know … (Participant 20, 28 years old).

Women also expressed the reality of the need to take PrEP as a type of wake-up call, reminding them of their risk behaviors and the role that a preventive tool like PrEP can play. This serves for some as a motivating factor, but for other women, this was as a deterrent and reflected the way substance use and self-care do not typically coincide. As one woman states:

If I used a needle, I probably would take [PrEP] every day, but who’s thinking of that one? I mean not wanting to take it, the drug obviously is not going to make you want to take it because you never remember like, why would I take this? That’s why so much HIV is out there. (Participant 36, 27 years old).

### Theme 3: perceptions of PrEP use by sexual partners

Women were moderately supportive of PrEP use by their sexual partners. This sentiment was often related to partner PrEP utilization as a possible indication of infidelity or partner engagement in high-risk substance use and/or sexual behaviors. Many women expressed partner PrEP use as a signal of deceitful behavior, as shared by one woman who stated:

If my partner was doing something like [sharing needles] I’d be pretty upset that he was out there …. He’ll go out and just hang out with his friends and there’s a thousand people and if one of you do it, they all do it and they’re sharing a fucking needle and then you never know. Just take [PrEP]. (Participant 36, 27 years old).

For many women in current sexual relationships, there was a common perception of mutual monogamy, but partner PrEP use could be a signal of infidelity. When interview questions probed about partner PrEP utilization, women would often wonder about the motives of their partner, as explained by one woman who shared:

I would question why they were taking it if they were just sexually active with me …. If we’re monogamous, there’s no reason for you to take it because if I’m having sex with just you and you’re just having sex with me. Why take it? … However, if they would like to, go ahead. By all means, protect yourself. (Participant 35, 30 years old).

Upon further probing, women who expressed suspicion and mistrust of a partner using PrEP would often follow up with a statement that they would not pass judgement on a partner indicating that it was “his business” to choose to take PrEP:

I think it’s smart, but at the same time, I would hope that you weren’t fooling around on me …. If I was with somebody, and they decided they wanted to get that medication, I would be all for it. If that’s something they want to do, I’m not going to stop them because they’re their own person. I let them do whatever they want because at the end of the day, they’re going to make their own decisions. (Participant 31, 24 years old).

Although women were moderately supportive of PrEP use by their sexual partners, they often appeared to have mixed feelings about partner PrEP use. This ambivalence demonstrated their fluctuating thoughts on the understanding of how partner PrEP utilization influenced their own personal HIV risk perceptions. As shared by one woman:

I’d be supportive as opposed to …. If he made a mistake or whatever and went out, I can’t blame him. So if he went out and had sex with somebody, of course, it would break my heart. If he went out and didn’t protect himself with a condom or something and was with another girl, and then told me, ‘Oh, now, I’m taking this PrEP stuff then fine’. Of course, I’d rather you be responsible and take something than to hide it and then have me find out 6 months later that because I gave you a blowjob or something, that all of a sudden, I have HIV. (Participant 9, 36 years old).

The issue of wavering trust was a common sentiment when women explored the idea of partner PrEP use. They expressed a hesitation where, on the one hand, they were guarded about the concept yet, on the other hand, supportive of the notion that a partner would take proactive behaviors to protect oneself. This support was often a reassurance that the partner was seeking out preventive measures to prevent HIV acquisition.

Some participants welcomed partner PrEP use as a potential avenue to expand into concurrent use, whereby both individuals would agree to initiate PrEP. Women expressed the notion that co-use of PrEP could be a bonding experience that might, in fact, strengthen their relationship and prioritize mutual safety. As shared by one participant:

If you feel that’s what you want to do, go ahead. More kudos to you. I mean, I wouldn’t be against it, not at all, because that’s somebody being safe. And maybe if I had a partner that takes PrEP, maybe I would, too. It could be like our daily thing. Let’s take our PrEP. (Participant 14, 29 years old).

## Discussion

This qualitative project is among the first set of studies to explore HIV risk perception and PrEP attitudes among women with OUD and provides an extension of the research base by focusing on justice-involved individuals. Our findings have unique value and relevance given that women account for nearly one in every five HIV diagnoses in the United States [33]. Findings from this study are critical for future PrEP implementation efforts among women with OUD, as well as those experiencing justice system involvement.

In this sample of cisgender women with OUD recruited from a specialized opioid court program, PrEP awareness was present, yet knowledge was limited; this finding is consistent with prior work among women [34, 35]. In addition, participants reported rates of bacterial STIs and hepatitis C higher than the general population [36] indicating increased HIV risk. Public health partnerships for HIV prevention can consider the justice system as a means to expand on harm reduction approaches [37]. For example, opioid court programs can be viewed as windows of opportunity to address not only OUD but also HIV prevention, including PrEP. The integration of harm reduction services into the justice system could aid in reducing risks related to HIV and hepatitis C [28, 30]. With the vast growth of opioid intervention court programs across the country [38], incorporating activities connecting infectious disease prevention with current services such as medication-assisted treatment is imperative. Our findings echo those of prior studies regarding a need for informed integration of harm reduction with HIV prevention services [39, 40].

Consistent with prior research [41–43], low perceived HIV risk was an overarching theme in our findings. Women did not perceive themselves to be at risk of contracting HIV and reported low PrEP interest and motivation. However, prevalence of bacterial STIs in our study sample was higher than the general population and signals a possible indicator of HIV risk; yet, many women did not connect sexually transmitted infection acquisition as a behavioral risk factor for subsequent HIV infection. Such miscalculated HIV risk perceptions may contribute to missed opportunities for PrEP engagement [44]. This disconnect is a growing problem, as those individuals eligible for PrEP may be least likely to seek preventive screening [35].

Barriers to PrEP initiation included commonly found obstacles in prior studies among women, such as side effects and adherence concerns [40, 45, 46]. Participants’ beliefs regarding facilitators to PrEP uptake were often represented in retrospective and prospective terms. For example, women discussed PrEP initiation in the hypothetical future where they may find themselves re-engaging in condomless sex with new or multiple partners, return to commercial sex work, and relapse scenarios where they envisioned PrEP as a valuable tool for HIV prevention. In light of the heightened risk of return to injection drug use, women’s perceptions of PrEP held a temporal element and offers a window of opportunity to allow for a consideration of PrEP as a means to be prepared for an unpredictable future [47].

PrEP interest and motivation were impacted by various factors influencing the decision to consider PrEP initiation or comfort with sexual partner use, as demonstrated in the thematic findings. Given findings from quantitative studies on gender norms and power differentials [48], it is plausible that women may disregard personal PrEP uptake for fear that the notion of mentioning it to sex partners may contribute to a partner’s suspicion of infidelity. This could influence a woman’s ability to use PrEP safely and reliably. In addition, women discussed their often overlapping IDU and sexual partnership networks which heighten their risk for HIV and elevate their need to consider PrEP as a prevention strategy. However, personal HIV risk assessment may underestimate actual risk, which has implications for PrEP uptake.

Interestingly, study participants were generally subdued in their perceptions of sexual partners’ use of PrEP. This finding was highlighted by perceptions that partner uptake of PrEP could signal infidelity, consistent with prior studies exploring the influence of partner mistrust on the uptake of HIV prevention strategies [49–52]. Of note, PrEP provides a clear benefit in that women can take the medication without a partner’s knowledge resulting in a layer of HIV prevention in circumstances where a woman’s partner may reject condom use, or due to fear of ensuing violence [53]. These realities can be powerful motivating factors for women which may tip the balance in favor of PrEP use as a new stratum of protection against HIV transmission. It offers a type of risk reduction method different from other preventive strategies which are independent of the need for partner cooperation or approval, as may be the case for condom use negotiation. PrEP provides an innovative opportunity that is in the hands of the user for control.

As found in several previous studies, the role of substance use is one of the most prevalent links between HIV risk and justice involvement [28, 37, 54, 55]. The criminal justice system is a growing site for HIV prevention interventions outside of the typical health care environment, such as emergency departments or clinics. Non-conventional sites, such as courts and prisons, have been utilized for various forms of HIV/hepatitis C prevention interventions [37, 56] and it is plausible that future programing will incorporate PrEP eligibility screening, HIV testing, and other linkages to community-based care, treatment, and prevention. Such risk reduction programming could be delivered efficiently and effectively in court settings.

### Clinical and practical implications

Due to a complex combination of biological, behavioral, and sociostructural dynamics, women who inject drugs are at greater risk of HIV compared with their male counterparts [28, 57, 58]. However, women are excluded from PrEP conversations. For example, currently only Truvada® (emtricitabine and tenofovir disoproxil fumarate) is approved for PrEP among women. Descovy® (emtricitabine and tenofovir alafenamide) is indicated for men and transgender women who have sex with men, but not women as they were not included in its clinical trials. Consequently, there is a growing need for gender-specific interventions targeting women at risk for HIV, including women with OUD. There is also concern that current PrEP prescribing guidelines may inadvertently disqualify women at risk for HIV [59].

PrEP marketing materials may not be considered broadly representative of the multiple audiences who are eligible for PrEP, including women or people who use drugs. To date, most PrEP outreach efforts in the United States have focused on men who have sex with men. A concerted public health approach can utilize multi-level efforts to educate the general community, potential candidates, and health care providers about PrEP [60]. However, outreach is directly related to accessibility and affordability. A recent agreement between the U.S. Department of Health and Human Services and Gilead Sciences, Inc. to donate PrEP to 200,000 persons per year who are at risk for HIV and uninsured, is anticipated to lessen the health care access gap for potential PrEP users [61]. For women with OUD in particular, prevention messaging should indicate that PrEP is efficacious for HIV transmission risk via needle-sharing as well as sex. In addition, the critical element of user control should be highlighted; condom negotiation with male sex partners adds a layer of complexity with cooperation with sexual partners (especially for women experiencing intimate partner violence, sex work, and sexual assault) while PrEP is a fully user-controlled prevention tool. Such autonomy is advantageous relative to other risk reduction strategies, such as condom use.

In 2016, the CDC released a report of 220 counties considered vulnerable to HIV and hepatitis C dissemination among PWID who were at risk due to intravenous drug use [7]. Public health concerns about HIV transmission among PWID were renewed following a recent HIV cluster identified by the Massachusetts Department of Public Health, with nearly half of the cases being female [62]. Given a series of additional outbreaks [9], the CDC has issued a comprehensive guide for state and local health departments in managing HIV and hepatitis C outbreaks among PWID, with recommendations for outbreak preparedness, detection, investigation and response [63]. Rather than wait until the next HIV cluster occurs, proactive attempts to simultaneously address OUD alongside HIV and hepatitis C are of critical importance for public health.

In light of study findings, various harm reduction strategies can be considered with a public health lens. First, recent HIV clusters among PWID have demonstrated that experiences with the criminal justice system were relatively common [9]. A future outbreak poses a key juncture to reconsider linkages between correctional health and public health systems, such as utilizing well-established support service models for people at risk of acquiring HIV [64]. While justice system programs that integrate OUD with HIV or hepatitis C testing are relatively uncommon, a recent systematic review concluded that joining together treatment programs with infectious disease screening can be effective [65]. Such collaborative harm reduction approaches lead to the development of stronger, more effective interventions that dually target OUD and HIV/hepatitis C prevention, and can extend to a meaningful expansion into a larger discussion into substance use disorder treatment facilities and syringe exchange programs [9].

### Limitations

As with all studies, our qualitative study has limitations to consider when interpreting our findings. Study participants were exclusively cisgender female and primarily White, recruited from one opioid intervention court program in a Northeastern city. In addition, as our study included individuals engaged in this specialized court program, we did not interview women who may be more socially isolated, less involved in health care and harm reduction services, and non-criminal justice involved individuals who may experience different patterns of HIV risk. Thus, their voices are not reflected in this study. Consequently, future studies could consider innovative strategies to engage individuals both in and outside of the justice system. Lastly, our study was based on self-report. Future projects could consider an exploration of other potential barriers to PrEP utilization among women, such as health care access, mistrust of the healthcare system, interpersonal violence, and power differentials with partners, as well as reviewing records for objective verification of self-report, given the complexity of medical and legal experiences.

## Conclusions

Our study findings provide meaningful insight for the development of future programming efforts to optimize PrEP uptake among justice-involved women with OUD. This is of particular interest given the increased HIV risk for women in the justice system. For PrEP implementation programs among justice-involved women and women with OUD, increasing awareness and accurate estimates of HIV risk are key points to consider. The promise of PrEP should involve all individuals who meet eligibility criteria for this biomedical intervention that can help prevent transmission and ultimately lead to a reduction in HIV incidence. Courts may provide a venue to offer patient education opportunities and provider assessments of patients’ knowledge, attitudes and receptiveness to a potentially life-saving medication.

## Data Availability

The data collected during the present study are not publicly available due to the protection of human participants’ confidentiality. The participant consent form stated that the data would not be shared with individuals outside the research study team.

## Abbreviations

HIV: Human Immunodeficiency Virus
IDU: Injection Drug Use
OUD: Opioid Use Disorder
PrEP: Pre-Exposure Prophylaxis
PWID: People Who Inject Drugs
